# Comparative Therapeutic Effects of Plant-Extract Synthesized and Traditionally Synthesized Gold Nanoparticles on Alcohol-Induced Inflammatory Activity in SH-SY5Y Cells In Vitro

**DOI:** 10.3390/biomedicines5040070

**Published:** 2017-12-15

**Authors:** Ashok K. Singh

**Affiliations:** Department of Veterinary Population Medicine, College of Veterinary Medicine, University of Minnesota, Twin-Cities campus, St Paul, MN 55108, USA; singh001@umn.edu

**Keywords:** ethanol, gold nanoparticles, plant extracts, kudzu root, edible gum, cell viability, apoptosis, necrosis, lactate dehydrogenase, lipid peroxidase

## Abstract

The present study describes potential beneficial and adverse effects of plant-extract synthesized gold nanoparticles (AuNPs) on ethanol toxicity in SH-SY5Y cells. Although kudzu root extract (K), edible-gum extract (G), alone or in combination (KG), reduced Au^3+^ into AuNPs, the extract’s composition and the reaction temperature determined their size (AuNP_KG(90<50<37)_ << AuNP_K__(90,50<37)_ < AuNP_G__(90<50)_; the subscript KG, K, or G is extract identification and numerical vales are reaction temperature in Celsius) and biological properties (AuNP_KG__(90,50>37)_ << AuNP_K__(90,50>37)_ < AuNP_G__(90,50)_). The surface of each AuNP contained the extract’s active ingredients, that were analyzed and confirmed using laser desorption ionization (LDI)) and low-matrix laser desorption-ionization (_L_MALDI). AuNP_KG-50_ was (i) least toxic to SH-SY5Y cells, but most effective in suppressing the adverse effects of ethanol on SH-SY5Y cells, and (ii) more effective than a combination of free kudzu and gum extracts. The beneficial and adverse effects of AuNPs may have been modified by the formation of proteins corona. This study provides a proof of concept for possible application of plant-extract synthesized AuNPs in mitigating ethanol toxicity.

## 1. Introduction

Alcohol use disorder, also known as alcoholism, is a pattern of alcohol use involving problems controlling one’s drinking, being preoccupied with alcohol, continuing to use alcohol even when it causes problems, having to drink more to get the same effect, or having withdrawal symptoms when one rapidly decreases or stops drinking. Untreated alcoholism devastates families, depresses economic vitality, and burdens the country’s health care systems, costing the United States over $249 billion annually [1,2,3,4]. Although possible mechanisms underlying the etiology of alcoholism are not fully understood, critical roles for opiate, gamma aminobutyric acid (GABA), serotonin, N-Methyl-D-Aspartate (NMDA), α-amino-3-hydroxy-5-methyl-4-isoxazolepropionic acid (AMPA) and kainate receptors have been implicated [5,6,7]. Consequently, Food and Drug Administration (FDA) has approved four drugs for treatment of alcoholism: naltrexone (an opiate antagonist), acamprosate (GABA receptors), disulfiram (acetaldehyde dehydrogenase inhibitor), and topiramate (AMPA/kainate receptor antagonist), that can be used alone or in combination with psycho-behavioral therapies and counseling [8]. Many other receptor-based drugs are being developed for future use [9]. The current pharmacotherapy, although effective, has certain disadvantages:

(i) Pharmacotherapy is a one-size-fits-all approach of addiction treatment that has not been largely successful, possibly because alcoholics constitute varying subtypes with differing biological and psychosocial contributions to the disease [10].
(ii) Alcoholism is a multifaceted disorder associated with neurochemical heterogeneity and behavioral complexities, but current and prospective anti-alcoholism drugs selectively target specific receptors or transporters [8,9]. Therefore, patients may have to take many pills daily for comprehensive protection/treatment of alcoholism. This may be partially responsible for the patients’ non-adherence to pharmacotherapy regimens.
(iii) The current therapeutic drugs exhibit poor bioavailability, serious side effects and probability of development of addiction (for benzodiazepines). Therefore, there is an urgent need to develop potent, effective and safer medications to treat alcohol-related disorders.

Currently, following alternative treatment approaches may constitute novel broad-spectrum pharmacotherapies for alcoholism and addiction.

- Herbal Medications: Although many Asian countries have used medicinal plants such as *Tabernanthe iboga*, *Panax ginseng*, *Salvia miltiorrhiza*, *Hypericum perforatum*, *Pelargonium graveolens*, *Lippia citriodora*, *Punica granatum*, *Morinda citrifolia* L., *Mirabilis jalapa*, *Aloysia triphylla*, etc., to treat addiction for centuries, only recently has the West begun to understand their pharmacological possibilities and clinical applications in alcoholism treatment [9,10,11]. Despite large volume of research data in support of medicinal plants’ anti-alcoholism effects, their clinical applications are hindered by poor solubility and bioavailability of the active ingredients, inability to cross the blood–brain barrier, and high variability [12]. Early work by Benlhabib et al. [13,14] showed that *Pueraria lobata* (kudzu root) contains three major isoflavones, puerarin (PU), genistein (GE), and daidzein (DE), exhibiting the following pattern: PU >> GE > DE. Either an aqueous extract of kudzu root or purified PU alone reduced (1) alcohol consumption (50% suppression) without affecting water intake, and (2) severity of the alcohol withdrawal symptoms in of alcohol-preferring rats when administered orally. In the studies where the isoflavones were given over the course of several days, maximal suppression of alcohol intake occurred in 2 to 3 days [14].
- Traditionally Synthesized Nanoparticles: Development of nanoparticle (NP)-based pharmacotherapy is a promising development in diagnosing and designing personalized treatment of addiction and other diseases [15]. Studies have used colloidal gold and silver NPs, functionalized with multiple pharmaceuticals and other active ligands, such as a blood–brain barrier permeant peptide, in treatment of alcoholism [16,17]. NPs, because of their unique properties, may circumvent the disadvantages of current pharmacotherapy discussed above and/or listed below [18,19]. Some of the advantages of NPs are (i) improved bioavailability and therapeutic efficacy; (ii) multiple drugs loaded in a single nanocarriers, resulting in improved compliance because patients will not have to take multiple pills; (iii) on-demand drug release—nanocarriers may be designed to release drugs as needed via external (ultrasound) or internal (pH or selected enzymes) cues. However, the traditionally synthesized gold and silver NPs have some disadvantages: they require stabilization to prevent rapid aggregation, difficult to functionalize with certain ligands, and undergo defunctionalization, releasing toxic NPs. Since the NPs contain the FDA approved drugs listed above, they thus exhibit the same limitations listed above for pharmaceutical preparation.
- Plant Extract Synthesized Nanoparticles: Earlier studies have described “green” synthesis of gold and silver NPs using plant extracts that are environment friendly, cost effective, easily scaled up for large scale syntheses of nanoparticles, and do not require stabilizers such as polyethylene glycols [20,21,22,23,24,25,26,27,28,29,30,31,32,33,34]. Most importantly, the “green” nanoparticles may retain the therapeutic potency of the plant and the unique properties of NPs. The key problems associated with the “green” NPs are lack of (i) methodology to identify the surface ligands; (ii) dose-response studies, and (iii) established therapeutic doses.

The overall goal of this study was to synthesize and characterize gold nanoparticles (AuNPs) using aquatic extract of kudzu root with or without edible gum. Kudzu root that has been shown to possess potent anti-alcoholism properties [33,34], while edible gum improves the quality of the NPs [35]. The hypothesis was that AuNPs synthesized with combinations of kudzu root and gum extract (spiked with an internal standard) retain the chemical composition of the extract and improve its therapeutic effects against ethanol toxicity in SH-SY5Y cells. The specific aims were to (1) characterize the AuNPs; (2) identify and quantify the surface ligands; (3) determine the AuNPs uptake into the SH-SY5Y cells and AuNP-protein interactions; and (4) assess the beneficial and adverse effects of AuNPs in SH-SY5Y cells. The methodologies were improved by including (i) an internal standard (4d-daidzein) in the extract used for synthesis of AuNPs preparations that allowed extract standardization, and (ii) laser desorption ionization (LDI) and low-matrix assisted-LDI (_L_MALDI) for analysis of AuNP surface ligands. The preliminary studies indicated that inclusion of “gum” in the reaction medium improved the AuNP’s synthesis by kudzu root extract. 

## 2. Materials and Methods

### 2.1. Nanoparticle Synthesis and Characterization

#### 2.1.1. The Traditional Synthesis

AuNPs were synthesized from AuCl_3_ using synthetic reducing agent, as described by Singh et al. [15].

#### 2.1.2. Plant-Extract Based Synthesis

Extract preparation: Commercial, pre-calibrated, kudzu root extract powder (20 g, Sigma Chemicals, St Louis, MO, USA) and 10 g Gum Arabic (Sigma Chemicals) were mixed in 100 mL double distilled water (DDW), and incubated under stirring 50 °C for 4 h. The mixture was filtered through a 0.45 μm filter (Millex^®^ polyvinylidene difluoride; EMD Millipore/Sigma, St Louis, MO, USA). The composition of the filtrate was analyzed as described previously [13,14]. The filtrate was frozen dried and stored at −80 °C for later use.

AuNP synthesis: In a 250 mL flask, 1 mM AuCl_3_ was mixed with 50 mL of filtered extracts (kudzu, gum, or mixture of the two) and 0.1 mL (1 mg/mL stock) of the internal standard (4d-daidzein) and heated to 37, 50, and 90 °C, which was maintained for 4 h (appropriate concentrations, temperature, and time were determined in a preliminary study). The UV-Vis (ultraviolet- visible spectrum was measured at 500 to 600 nm, to assess NP formation and detect the corresponding excitation bands. After incubation (the preliminary study showed that 4 h of incubation at 50 °C, or 2.5 h at 90 °C was sufficient for conversion of AuCl_3_ to AuNPs, 5 to 10 nm), the mixture was centrifuged, and the AuNP pellets were collected and washed 3× with distilled water. 

### 2.2. Nanoparticles Were Characterized by Measuring the Following Indices 

#### 2.2.1. UV–vis Spectrum

Synthesized AuNPs were confirmed by sampling the aqueous component of different time intervals and the absorption maxima was scanned by UV-VIS spectrophotometer at the wavelength of 300–800 nm on Perkin-Elmer Lambda 25 spectrophotometer (Perkin-Elmer, Walthan, MA, USA). 

#### 2.2.2. FT-IR Spectrum

AuNP samples were dried and ground with KBr (potassium bromide) pellets for Fourier transform infrared (FTIR) measurements. The spectrum was recorded in the range of 4000–400 cm^−1^ using a Varian FTS 1000 FT-IR Spectrometer (Varian Inc. Palo Alto, CA, USA) in the diffuse reflectance mode, operating at a resolution of 4 cm^−1^.

#### 2.2.3. Surface Topology by Transmission Electron Microscope (TEM)

Morphology and size of the gold nanoparticles were investigated by (TEM) images using Hitachi model HF-2000 field emission transmission electron microscope (Hitachi, Tarrytown, NY, USA) with a resolution of 0.1 nm. A thin film of the sample was prepared on a carbon coated copper grid by dropping a very small amount of the sample on the grid and drying under a lamp.

#### 2.2.4. Identification of AuNPs and the Surface Functional Groups

Laser desorption ionization (LDI) without matrix or low-matrix-assisted LDI time-of-flight (TOF) mass spectrometer (MS) (Bruker Autoflex III Smartbeam MS, Bruker, Billerica, MA, USA) equipped with a 355 nm Nd:Y_3_Al_5_O_12_ laser were used in characterization of AuNPs and the functionalized groups. The nanoparticle preparations (1 µL) were mixed with 1 μL of (i) 50% acetonitrile (without matrix) and 0.1% trifluoroacetic acid (TFA), and (ii) 3 mg (for low-matrix) and 20 mg (for full matrix) of α-cyano-4-hydroxycinnamic acid (CHCA) solution. The mixtures were crystallized onto the LDI-disc. MS experiments were done in positive mode on a reflectron-type (20.92 kV) time-of-flight (to acquire a single spectrum, 200 laser shots were fired at a frequency of 100 Hz at 35–40% of the full laser power). In all cases, multiple spectra were averaged to obtain the reported data. 

### 2.3. Beneficial Efficacy and Adverse Effects of AuNPs

**Figure 1:**
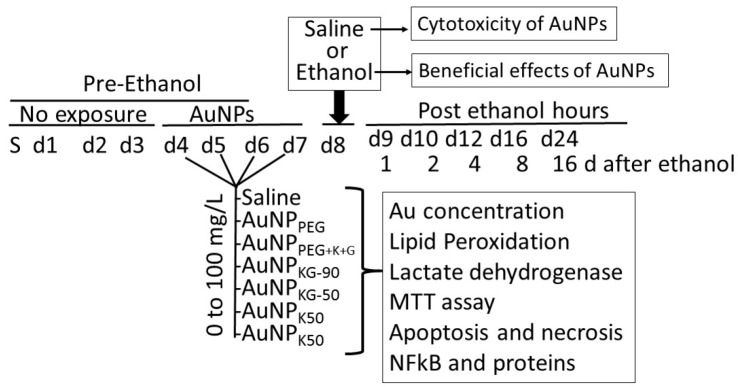
Experimental protocol. SH-SY5Y cells were expanded in growth media for 3 days. On day-1, 4, 3 × 10^7^ cells were transferred into each well, and incubated for 4 days in media containing different doses of gold nanoparticles (AuNPs) (AuNP positive (Ap) cells) or matrix alone (AuNP negative (An) cells). At day 8, cells were washed and incubated in media containing 54 mM (final concentration) ethanol (Ap ethanol exposed (ApEp), or An ethanol exposed (AnEp) cells) or the matrix alone (AuNP and ethanol negative (AnEn), or Ap ethanol negative (ApEn) cells) for 24 h. Then, cells were washed and incubated in media alone. At different time intervals after ethanol exposure, cells were harvested and analyzed for AuNP internalization, AuNPs’ adverse effects, and modulation of ethanol toxicity. PEG: polyethylene glycol; K: kudzu root extract; G: gum extract; KG: combination of kudzu root and gum; MTT: 3-(4,5-dimethylthiazol-2-yl)-2,5-diphenyltetrazolium bromide; NFκB: nuclear factor kappa B.

Beneficial and adverse effects of the AuNPs were investigated using SH-SY5Y neuronal cell-lines. For culture, the vials containing SH-SY5Y cells were thawed rapidly in a 37 °C water bath, and the contents were transferred in a centrifuged tube containing 9.0 mL complete culture medium (1:1 mixture of Eagle’s Minimum Essential Medium and F12 Medium mixed with fetal bovine serum, final concentration 10%). The cells were centrifuged at 125× *g* for 7 min, and the pellets were suspended with the complete medium (pH 7.0 to 7.6). The cells where incubated at 37 °C in 5% CO_2_ in air atmosphere for 3 days. Then, the cells were plated at a density of 10^7^ cells in CytoOne filter cap flasks and cultured for 3 days. Then, the media was replaced with the one containing either different concentrations of AuNP preparations (AuNP-positive cells) or the matrix alone (AuNP-negative cells) that continued for 4 days (day 4 to day 7). On day 8, cells were exposed for 24 h to either 50 mM ethanol (ethanol-positive cells) or saline (ethanol-negative cells) as matrix (Figure 1). This yielded the following cell groups: (1) AuNP-negative/ethanol-negative (An/En); (2) AuNP-negative/ethanol-positive (An/Ep); (3) AuNP-positive/ethanol-negative (Ap/En); and (4) AuNP-positive/ethanol-positive (Ap/Ep) cells.

At day 9, the cells were incubated in AuNP and ethanol free culture media for another 16 days. At predetermined times (day 9 to day 25 (i.e., day 0 days post ethanol), cells were harvested and processed for the following:

(i) Determination of internalized AuNPs by measuring intracellular Au concentrations (iAuCs): the atomic absorption spectrometric method described by Pedersen and Graabaek [36] and Benlhabib, et al. [13,14] was used for analysis of Au in tissue samples. In brief, the cells were harvested, treated with trypsin, etched with potassium iodide (KI) and iodine (I_2_) at 1:6 ratio (0.34 mM I_2_) to remove the surface AuNPs [37], washed with plasma buffered saline, and mixed with 5 mL of aqua regia, and digested for 24 h. The digest was diluted 1:1 with a solution of 2 ppm yttrium in dilute nitric acid, serving as an internal standard. The instrument was calibrated using a solution of 1 ppm Au and 1 ppm yttrium in 50% aqua regia. The samples were introduced in a segment flow mode of a flameless atomic absorption spectrophotometer (Beckman model 485, Beckman, Indianapolis, IN, USA). Au concentration was determined using a calibration curve. The brain Au concentrations were normalized to total tissue weight.
(ii) Lactate dehydrogenase release assay [38]: 50 μL of clear cell media was mixed with the reconstituted 2× LDH assay buffer (223 mg 2-p-iodophenyl-3-p-nitrophenyl-5-phenl tetrazolium chloride, 57 mg N-methylphenazonium methyl sulfate, 575 mg nicotinamide adenine nucleotide (NAD), and 3.2 g lactic acid in 480 mL 200 mM Tris buffer solution, pH 8.0). The mixture was gently shaken for 30 seconds and incubated in dark for 10 to 30 min at room temperature. Reaction was stopped with 50 μL of Stop Solution (1 M acetic acid), mixed, and absorbance was measured between 490–520 nm.
(iii) Lipid peroxidase: the cells (3.6 × 10^6^) were suspended in 2 mL of a solution containing 15% trichloroacetic acid, 0.25 N HCl and 0.5% thiobarbituric acid, and the samples were heated for 25 min in a boiling water bath. The samples were cooled and centrifuged for 10 min at 4000 rpm. The absorbance of the supernatant fraction was determined at a wavelength of 535 nm. An extinction coefficient of 1.56 × 10^5^ M^−1^ cm^−1^ was used to calculate the concentration of malondialdehyde. Values were expressed as pmol of MDA per mg protein.
(iv) Cell viability: the MTT (3-(4,5-dimethylthiazol-2-yl)-2,5-diphenyltetrazolium bromide) tetrazolium reduction assay kit from Millipore (Millipore Sigma, St Louis, MO, USA) was used to assess cell viability.
(v) Analysis of cells undergoing apoptosis and necrosis: cells were exposed to the AuNPs followed by ethanol as shown in Figure 1. At different time intervals after cessation ethanol, the cells were harvested and double stained with annexin V-FITC (An) and propidium iodide (PI), as described previously [15,38], and were subjected to fluorescence activated cell sorting (FACS) analysis using on a FACScan flow cytometer (Becton Dickinson, San Jose, CA, USA). All the experiments were performed in triplicate. Cells were classified as An^−^PI^−^ or An^+^ PI^−^ (normal cells), An^−^PI^+++^ (necrotic cells), An^++^PI^−^ (early apoptosis stage), An^+++^PI^+^ (late apoptosis stage).
(vi) NFκB activation by electrophoretic mobility shift assay (EMSA) [39]: cell cultures from in vitro studies or finely chopped tissue samples from in vivo studies were suspended in HEPES (4-(2-hydroxyethyl)-1-piperazineethanesulfonic acid) hypotonic buffer A (10 mM HEPES, pH 7.9, 10 mM KCl, 0.1 mM EDTA (ethylenediaminetetraacetic acid), 0.1 mM EGTA (ethylene glycol tetraacetic acid_, 1 mM Dm, 0.5 mM phenylmethylsulfonyl fluoride, and 10 pg/mL leupeptin, antipain, aprotinin, and pepstatin) for 15 min on ice. Samples were vortexed for 10 s with 0.6% Nonidet P-40, and centrifuged at 12,000× *g* for 60 s. The pellets containing nuclei were resuspended in nucleus buffer (20 mM HEPES, pH 7.9, 25% glycerol, 0.4 M NaCl, 1 mM EDTA, 1 mM EGTA, 1 mM dithiothreitol, 0.5 mM phenylmethylsulfonyl fluoride, and 10 pg/mL leupeptin, antipain, aprotinin, and pepstatin) and briefly sonicated on ice. For EMSA, nuclear extracts (10 pg of protein) were incubated in 25 µL of total reaction volume containing 20 mM HEPES, pH 7.9, 50 mM NaCl, 0.1 mM EDTA, 1 mM DTT, 5% glycerol, 200 pg/mL bovine serum albumin, and 2.5 pg of poly(d1-dC) for 15 min at 4 °C. The carboxytetramethyl-rhodamine (TMR)-labeled oligonucleotide (0.5 ng) was then added to the reaction mixture, and incubated for 20 min at room temperature.

RelA-p50 probe: TMR-AGT TGA GGG GAC TTT CCC AGG CAA-3 

Reference probe: TMR-TCA ACT CCC CTG AAA GGG TCC GTT-5

Gels were scanned on the Hitachi FMBIO II using 585 channels for the isotope probe.

### 2.4. Characterization of AuNP–Protein Interaction

To investigate the AuNP–protein interaction [40], different AuNP preparations were incubated with fresh serum (1:10 by volume) at 37 °C for different time intervals. Thereafter, an equal volume of sucrose solution (0.75 M) was carefully layered, and the samples were centrifuged at 10,000× *g* for 1 h to separate unbound proteins (remained in water phase) from the AuNPs (settled at the bottom). The pellets were collected and washed three times with isotonic saline to remove soft corona. The hard corona proteins were desorbed using CHAPS buffer (DNase 1 (100 U/mL), RNAse A (10 U/mL), CaCl_2_ (20 mM), MgCl_2_ (50 mM), Tris HCl pH 7 (100 mM), SDS 0.02%, ampholine (1%), CHAPS (0.8%), β-mercaptoethanol (1%), and urea (8 mM)). The mixture was centrifuged (15,000 rpm, 3 min) to separate the AuNPs (in the pellets) and the desorbed proteins (in the supernatant). The desorbed proteins were concentrated, mixed with bromophenol blue (0.5 µL) and run on 1-deminson gels. The separated proteins were staining with Coomassie dye or viewed for fluorescence under UV (ultraviolet) light.

For protein identification, the surface-adsorbed proteins were reduced with 10 mM dithiothreitol (DTT) at 37 °C for 1 h, and alkylated with 25 mM iodoacetamide (IAA) at room temperature for 50 min. For the digestion, the samples were incubated with porcine trypsin (Promega, Madison, WI, USA) for 8 h at 37 °C. The digested peptides were concentrated and purified in reverse phase C18 µcolumn, and eluted with the acid solution consisting of 10 mg/mL in 50% acetonitrile/milli-Q water (*v/v*), 0.1% trifluoracetic acid (TFA). 

An Agilent-1100 nanoflow LC system (equipped with a solvent degasser, nano-flow pump, and temperature controlled autosampler, a 7 Tesla Finnegan linear quadrupole ion trap Fourier transform (LTQ-FT) mass spectrometer and an LTQ-Orbitrap from Thermo Electron, Bremen, Germany) was used for nanoscale liquid chromatography tandem mass spectrometry of the digested peptides. Tryptic peptides were separated on 30 cm reverse phase column. 75 μm inner diameter (SGE analytical, Pflugerville, TX, USA) mounted on the nano-electrospray ion source at a flow rate of 250 nL/min and were separated over 30 min using a linear gradient of 10–40 (*v/v*) acetonitrile/0.5% (*v/v*) acetic acid). For MS, the applied voltage was 2.4 kV to the emitter. Full scan MS spectra (from *m*/*z* 300–1575) were acquired in the ion cyclotron resonance (ICR) mode with a resolution R = 25,000 at *m*/*z* 400 (after accumulation to a target value of 5 × 10^6^ in the linear ion trap). The ions monitored are listed in Table 1.

For immunostaining, the separated proteins were transferred onto a PVDF (polyvinylidene fluoride) membrane, blocked and incubated with rabbit anti-mammalian-IgG selective antibodies (reactivity to rat proteins were predetermined), followed by horseradish peroxidase (HRP)-conjugated goat anti-rabbit IgG antibodies. For detection, an HRP-conjugated secondary antibody enhanced chemiluminescence kits from Abcam (Cambridge, MA, USA) were used.

### 2.5. Statistical Analysis

Data were recorded as mean ± SD using Microsoft Excel. Statistical analysis was performed using ANOVA (analysis of variance) followed by Tukey’s Multiple-Comparison Test or *t*-test at <0.05 significance level.

## 3. Results

### 3.1. Nanoparticle Synthesis and Characterization

#### 3.1.1. Physicochemical Characterization

**Figure 2:**
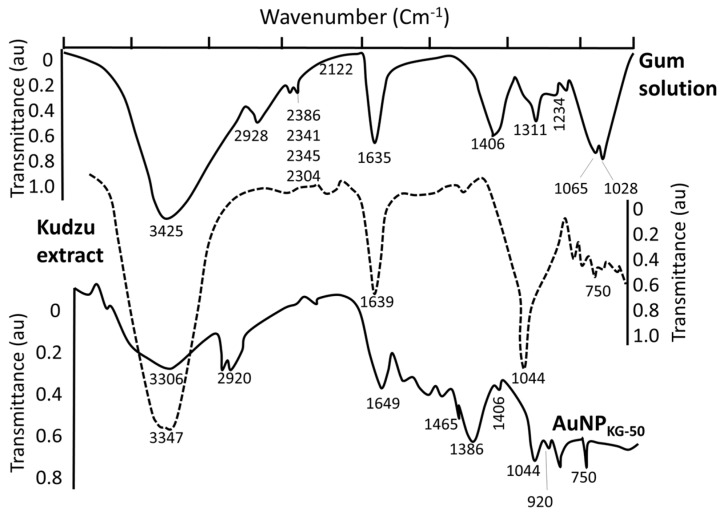
Typical FTIR spectra for AuNP_KG_, kudzu extracts, and gum solution.

**Figure 3:**
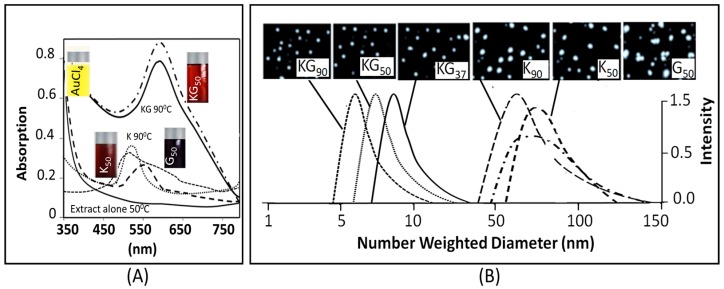
Optical absorption curves (**A**); size distribution and transmission electron microscopy (**B**) of different AuNP preparations. Abbreviations: K: kudzu, G: gum, and 37, 50, and 90: reaction temperature in °C.

AuCl_4_^−^ (a yellow colored aqueous solution) exhibited strong absorption at 217 nm, and a weak absorption at 287 nm. Exposure of AuCl_4_^−^ to reducing agents immediately changed its color from yellow to red/brown characterized by a strong absorption from 500–665 nm range, depending upon the nanoparticle size and synthesis procedure (Figure 2). A combination of kudzu and gum (KG) extract at 90 °C, 50 °C, and 37 °C yielded AuNPs with an average diameter of 8 nm, 12 nm, and 20 nm, respectively (Figure 2). The kudzu (K) extract alone at 90 °C, 50 °C, and 37 °C formed relatively larger AuNPs (diameter 30 to >50 nm). The gum solution (G) alone yielded particles >50 nm in diameter. The FTIR spectra of K extract and different nanoparticles are shown in Figure 3. The extract exhibited strong absorption at 2928, 1641, 1382, and 1069 cm^−1^, and weak absorption at 1456, 1156, and <500 cm^−1^. The AuNPs (i: AuNP_KG-50_, ii: AuNP_KG-95_, and iii: AuNP_K_) exhibited strong absorption at 3420, 1609, 1078, and 1069 cm^−1^, and weak absorption at 2920, 1456, 1383, 1156, 981, and <500 cm^−1^).

#### 3.1.2. The Surface Characterization

**Figure 4:**
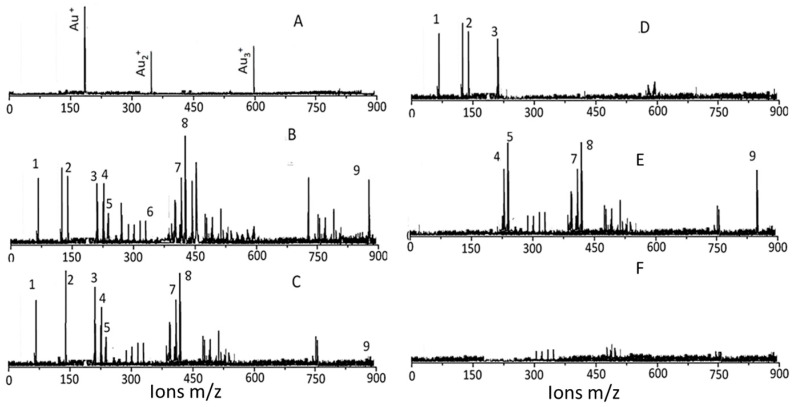
Mass spectral identification of surface ligands. (**A**) _L_MALDI that detects Au ions; (**B**) typical LDI (matric free) analysis of AuNP_KG-50_ synthesized by a mixture of kudzu root and gum; (**C**) typical LDI analysis of a mixture of kudzu and gum solutions; (**D**) typical LDI (matrix free) analysis of AuNP_G-50_ synthesized by a gum solution alone; (**E**) typical LDI (matrix free) analysis of AuNP_K-50_ synthesized by a kudzu extract alone; and (**F**) analysis of a blank solution. Peak identification 1: 73 *m*/*z* arabinose, galactose, fructose; 2: 147 *m*/*z* galactose; 3: 217 arabinose; 4: 225 *m*/*z* daidzein; 5: 229 *m*/*z* d4-daidzein; 6: 370 *m*/*z* not identified; 7: 417 *m*/*z* puerarin, 8: 429 not identified; 9: 894 kakkasaponin; Au^+^ 193 *m*/*z*; Au_3_^+^ 396 *m*/*z*.

Low-matrix MALDI detected Au^+^ (*m*/*z* 193), Au_2_^+^ (*m*/*z* 396), and Au_3_^+^ (*m*/*z* 589) (Figure 4A). The LDI-TOF MS detached the surface ligands for AuNP_KG_ (Figure 4B), free standards (Figure 4C), AuNP_G_ (Figure 4D), AuNP_K_ (Figure 4E), and blank (Figure 4F). The identity of fragment ions was confirmed by analyzing components of kudzu root extract (daidzein, d4-daidzein internal standard, puerarin, and kakkasaponin III) and gum solution (arabinose, galactose, and fructose). This suggests that, when nanoparticles are synthesized with plant extract, their components adsorb onto the surface. 

### 3.2. AuNP Internalization in Cells

**Figure 5:**
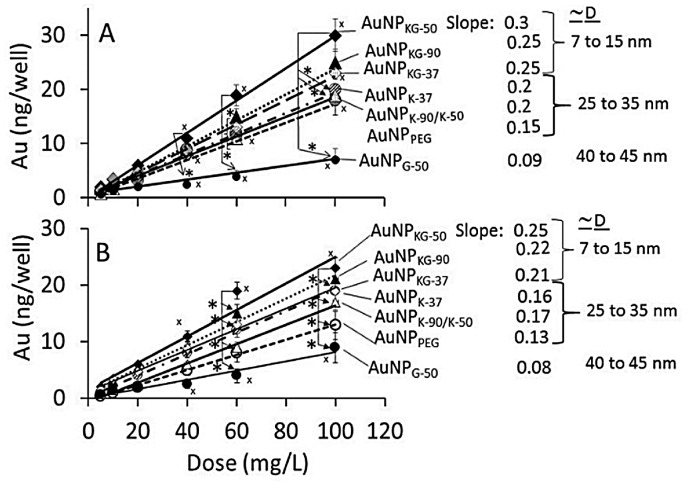
The dose (mg/L of AuNPs) versus response (intracellular Au concentration—iAuC) curves for internalization of different AuNPs at day 16 of cell incubation. (**A**) Ap/En cells, and (**B**) Ap/Ep cells. Values are mean ± SD. *: *p* < 0.05, significant, when compared with corresponding data from different groups, x: *p* < 0.05, significant, when compared with different dose values from the same group.

The dose-internalization curves for different AuNPs (measured as iAuC) were linear from 5 to 100 mg/L AuNP dose (Figure 5). The r^2^ for Ap/En and Ap/Ep cells ranged from 0.97 to 0.99, and 0.78 to 0.95, respectively, indicating greater variability in Ap/Ep cells. As shown below, the iAuCs were highest in cells exposed to AuNP_KG-50b_ and lowest in cells exposed to AuNP_G_. The slopes of the curve ranged from 0.06 to 0.1.

Ap/En cells: AuNP_KG-50_ > AuNP_KG-90,KG-37,K-37_ > AuNP_K-37,k-50,k-90,PEG+K+G_ > AuMP_G-50_

Ap/Ep cells: AuNP_KG-50_ > AuNP_KG-90,KG-37,K-37_ > AuNP_k-50,k-90_> AuNP_PEG+K+G_ > AuMP_G-50_

**Figure 6:**
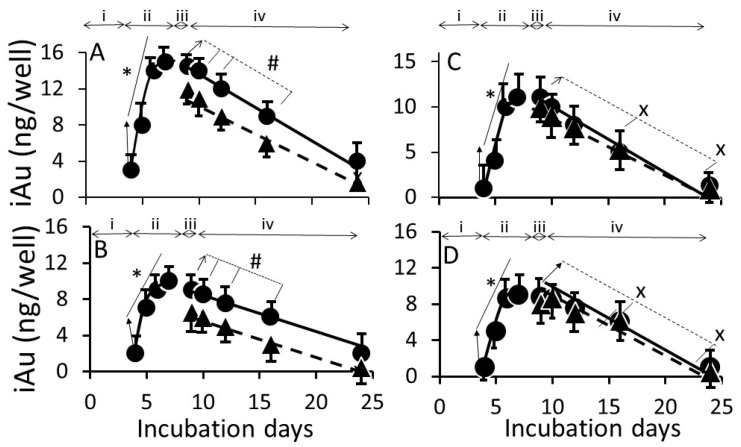
The time course of change in intracellular AuNP_KG-50_ (**A**), AuNP_K-50_ (**B**), AuNP_G-50_ (**C**), and AuNP_PEG+K+G_ (**D**) concentrations in Ap/En cells (solid line) and Ap/Ep cells (broken line). x: *p* < 0.05 when day 8 (before ethanol/matrix exposure) values were compared with post-ethanol values. #: *p* < 0.05 when corresponding Ap/En values were compared with corresponding An/Ep values. Please define *: *p* < 0.05, significant, when compared with the lowest value from the same group. Abbreviations: i: three days of cell growth (days 1 to 3); ii: four days of AuNP or matrix pretreatment (day 4 to day 7); iii: 24 h ethanol or saline (matrix) exposure (day 8) and iv: AuNP and ethanol free media for 16 days.

Figure 6 shows the time course of iAuC changes in cells harvested at (i) culture days 4 to 7 (days 1 to 4 of AuNP exposure); (ii) culture day 8 (24 h ethanol exposure); and (iii) culture day 9, 10, 14, 18, and 26 (16 days free of ethanol an NPs) as shown in Figure 1. From day 4 to day 7, iAuCs increased gradually and, at day 4, the iAuCs showed the following pattern: AuNP_KG-90_, AuNP_KG-50_ and AuNP_KG-37_ > AuNP_K-90_, AuNP_K-50_ and AuNP_K-37_ > AuNP_PEG_ and AuNP_G-50_. In Ap/En cells, a linear decrease in iAuCs occurred. The values at day 24 (16 days after discontinuation ethanol) ranged from 0 to 4 ng/well. In Ap/Ep cells, the post-ethanol iAuCs were significantly lower than those in corresponding Ap/En cells (Figure 6). The day 24 (16 days after discontinuation of ethanol) values for Ap/Ep and Ap/En cells did not differ significantly. 

### 3.3. Characterization of Surface Ligands in AuNPs Collected from Exposed Cells

**Figure 7:**
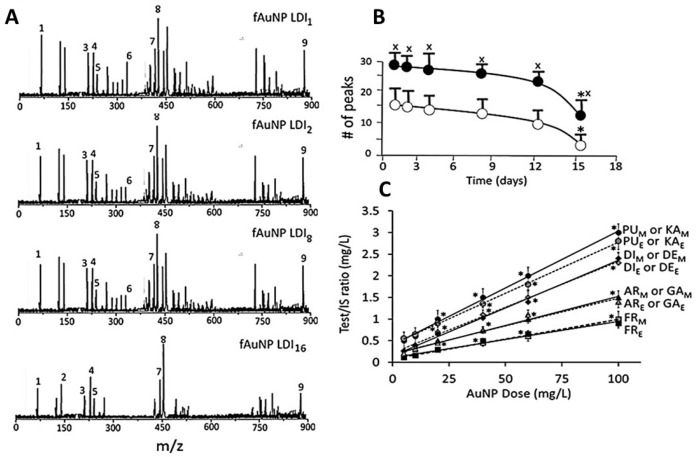
Identification of surface ligands on AuNPs collected from Ap/En and Ap/Ep cells. (**A**) the plant component peaks at 1 (fAuNP LDI_1_), 2 (fAuNP LDI_2_), 4 (fAuNP LDI_4_), 8 (fAuNP LDI_8_), and 16 (fAuNP LDI_16_) days after ethanol exposure; (**B**) Time course of change in total number of peaks adsorbed onto the AuNP surface. Total number of peaks decreased significantly at day 16 (filled circle: number of peaks in matrix exposed cells, open circle: number of peaks in ethanol exposed cells). (**C**) AuNP dose versus (ligand peak/IS peak) ratio plots for surface ligands in matrix exposed (subscript _M_) and ethanol exposed (subscript _E_) cells. Abbreviations: PU: puerarin, KA: kakkasaponin, DI: daidzin, DE_:_ daidzein, AR: arabinose, GA: galactose, FR: fructose. Values are mean ± SD. x: *p* < 0.05 when compared with corresponding open circle values, and *: *p* < 0.05 when compared with values from same group.

Figure 7 shows a typical time course and dose-response of change in surface ligands of intracellular AuNP_KG-50_ in Ap/En cells (Figure 7A—fAuNP_1_, fAuNP_2_, fAuNP_4_, fAuNP_8_, fAuNP_16_ represent 1, 2, 4, 8, and 16 days after ethanol exposure). Figure 7B shows time course of change in total number of peaks observed onto the AuNP_KG-50_ surface. The peak numbers remained unchanged for up to 8 days, but decreased significantly at day 16 after ethanol exposure. The Np/En values were significantly higher than the Ap/Ep values. This may be due to desorption and degradation of the ligands. Figure 7C shows a linear relationship between the AuNP_KG-50_ dose and the IS–peak area/ligand–peak area ratio. 

### 3.4. Beneficial and Adverse Effects of AuNPs

To evaluate the adverse effect of AuNPs alone, Ap/En cells were exposed to different concentrations of individual AuNPs for 4 days, the saline (matrix for ethanol) for 24 h. The cells were assayed for (i) viability using MTT metabolism assay; (ii) apoptotic and necrotic cells; and (iii) lipid peroxidase and lactate dehydrogenase activities. The beneficial effects were studied by incubating Ap/Ep cells with different doses of AuNPs for 4 days, followed by 50 mM ethanol, and then assayed as above. 

#### 3.4.1. Cell Viability

**Figure 8:**
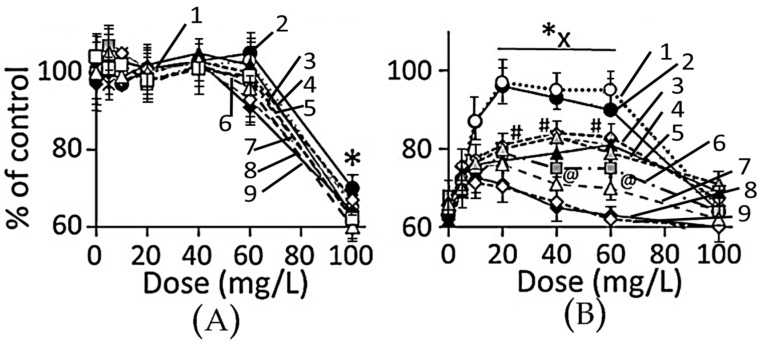
(**A**) Plots of dose (AuNP mg/L) versus viable Ap/En cells (% of control) at day 16. A 5 to 60 mg/L dose of all AuNP exhibited comparable response. Only 100 mg/L dose caused significant decrease in viable cell enumeration. Values are mean ± SD; (**B**) Plots of dose (AuNP mg/L) versus viable Ap/Ep cells (% of control) at day 16). Different AuNPs yielded variable response, but maximum protection was provided by AuNP_KG-50,-90_ at 20 mg/L dose. Values are mean ± SD. *: *p <* 0.05 when compared with An/En response and x, # or @: *p* < 0.05, when viability of Ap/Ep cells exposed to a given dose of and AuNP compared with viability of Ap/Ep exposed to the same dose of other AuNPs. 1: AuNP_KG-90_, 2: AuNP_KG-50_, 3: AuNP_KG-50_, 4: AuNP_KG-37_, 5: AuNP_K-50,90_, 6: AuNP_PEG_+KG, 7: AuNP_KG-37_, 8: AuNP_PEG_, 9: AuNP_G-50,G90_.

Dose–response: in Ap/En cells, a 5, 10, 20, 40 or 60 mg/L dose of different AuNP preparations did not significantly affect, while a 100 mg/L dose of AuNPs caused 30% to 60% decrease in viable cells (Figure 8A, hour 16 data). Ethanol exposure of An/Ep and Ap/Ep cells significantly decreased cell viability (Figure 8B). A 5, 10, 20, 40 or 60 mg/L dose of different AuNP preparations protected (10% to 40% reduction in MTT response) against the ethanol-induced decrease in cell viability (AuNP_KG-50 or KG-90_ > AuNP_KG-37_ > AuNP_K-90,K-50_ > AuNP_K37_, AuNP_PEG+K+G_> AuNP_G-50 orG90_ or AuNP_PEG_). A 100 mg/L dose of all AuNP preparations either did not change, or augmented ethanol toxicity. This suggests that the 20 and 40 mg/L dose of AuNP_KG-50_ and AuNP_KG-90_ were most effective against ethanol toxicity in SH-SY5Y cells. 

**Figure 9:**
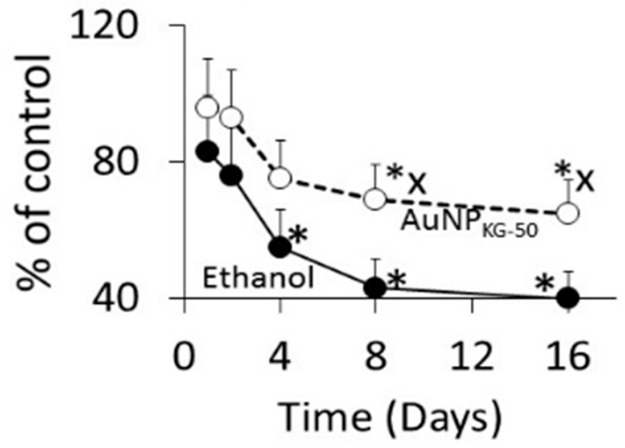
The time course of change in viability of Ap/En (broken line) and Ap/Ep (solid line) cell pre-exposed to a 20 mg/L dose of AuNP_KG-50_. *: *p* < 0.05 when exposed to day 0 values and x: *p* < 0.05 when Ap/En and Ap/Ep values were compared at each time interval.

Time course (Figure 9): at 1 and 2 days after cessation of ethanol exposure in AuNP_KG-50_-pretreated (Ap/Ep) or AuNP_KG-50_-negative (An/Ep) cells, about 70% of total cells were viable, and the values did not differ significantly between the two cell groups. Thereafter, the AuNP_KG-50_-pretreated values were significantly greater than AuNP_KG-50_-negative cells at all time intervals.

#### 3.4.2. Lipid Peroxidase and Lactate Dehydrogenase Activities

**Figure 10:**
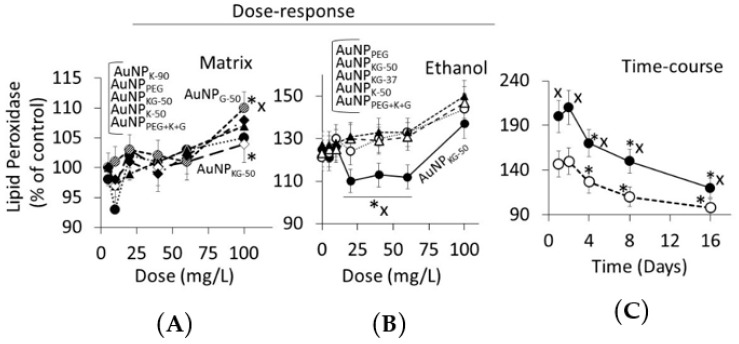
(**A**) Plots of dose (AuNP mg/L) versus intracellular lipid peroxidase activity in Ap/En cells (% of control) at day 16. A 5 to 60 mg/L dose of all AuNPs exhibited comparable response. Only 100 mg/L dose caused significant but variable increase in enzyme activity (AuNP_KG-50_ exhibited lowest, while AuNP_G-50_ exhibited highest activity in response to 100 mg/L dose). Values are mean ± SD; (**B**) Plots of dose (AuNP mg/L) versus intracellular lipid peroxidase activity in Ap/Ep cells (% of control) at day 16. Different AuNPs yielded variable response, but maximum protection was provided by AuNP_KG-50_ at 20 to 60 mg/L dose. Values are mean ± SD. *: *p* < 0.05 when Ap/En or Ap/Ep values were compared with An/En values. X, # or @: *p* < 0.05, when enzyme activity of Ap/Ep cells exposed to a given dose of and AuNP compared with enzyme activity of Ap/Ep cells exposed to the same dose of other AuNPs; (**C**) The time course of change in enzyme activity of Ap/En (broken line) and Ap/Ep (solid line) cell pre-exposed to a 20 mg/L dose of AuNP_KG-50_. *: *p* < 0.05 when exposed to day 0 values, and x: *p* < 0.05 when Ap/En and Ap/Ep values were compared at each time interval.

**Figure 11:**
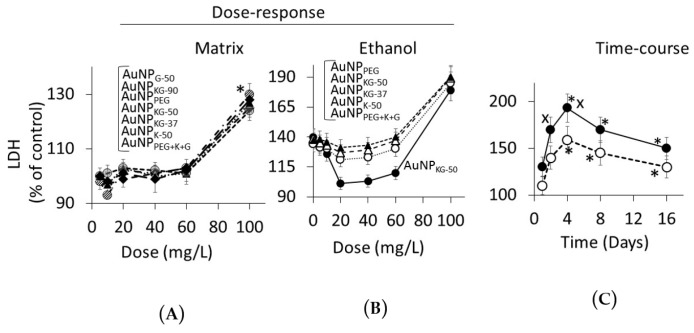
(**A**) Plots of dose (AuNP mg/L) versus lactate dehydrogenase (LDH) activity in Ap/En cells (% of control) at day 16. A 5 to 60 mg/L dose of all AuNPs exhibited comparable response. Only 100 mg/L dose caused significant in enzyme activity. Values are mean ± SD; (**B**) Plots of dose (AuNP mg/L) versus LDH activity in Ap/Ep cells (% of control) at day 16. Maximum protection was provided by AuNP_KG-50_ at 20 to 60 mg/L dose. Values are mean ± SD. *: *p* < 0.05 when Ap/En or Ap/Ep values were compared with An/En values, x: *p* < 0.05, when enzyme activity of Ap/Ep cells exposed to a given dose of and AuNP compared with enzyme activity of Ap/Ep cells exposed to the same dose of other AuNPs; (**C**) The time course of change in LDH activity of Ap/En (broken line) and Ap/Ep (solid line) cells pre-exposed to a 20 mg/L dose of AuNP_KG-50_. *: *p* < 0.05 when exposed to day 0 values, and x: *p* < 0.05 when Ap/En and Ap/Ep values were compared at each time interval.

Dose Response (Figure 10 and Figure 11): in Ap/En cells, a 5, 10, 20, or 40 mg/L dose of all AuNP preparations did not increase, a 60 mg/L dose slightly increased, and a 100 mg/L dose significantly increased the enzyme activities (Figure 10A for lipid peroxidase and Figure 11A for LDH). The enzyme activities (Figure 10A for lipid peroxidase and Figure 11A for LDH) showed the following patterns in Ap/En cells: AuNP_G-50_ and AuNP_K-90_ >>AuNP_PEG_ > AuNP_PEG+K+G_, AuNP_KG-50_, AuNP_KG-37_, and AuNP_KG-90_. In An/Ep cells or Ap/Ep cells, exposure to AuNP_G-50_, AuNP_K-90_, AuNP_K-50_, AuNP_KG-37_, AuNP_PEG_ and AuNP_PEG+K+G_ caused 125% to 155% increase in the enzyme activities (Figure 10B for lipid peroxidase and Figure 11B for LDH). In Ap/Ep cells, exposure to AuNP_KG-50_ or AuNP_KG-90_ (dose: 20, 40 and 60 mg/L) ethanol exposure did not significantly increased the enzyme activities (Figure 10B for lipid peroxidase and Figure 11B for LDH).). However, ethanol exposure of AuNP_KG-50_ pretreated (dose: 100 mg/L) cells significantly increased the enzyme activities. This suggests that a 20 to 60 mg/L dose of AuNP_KG-50_ or AuNP_KG-90_ protected Ap/Ep cells against ethanol toxicity.

Time course (Figure 10C for lipid peroxidase and Figure 10C for LDH): The two enzymes exhibited different time course of change in response to ethanol exposure. Lipid peroxidase activities were significantly higher at day 1 and 2, then decreased gradually to 130% of control at day 16. The ethanol-induced increase in peroxidase activity in An/Ep cells were significantly greater than the activity in Ap/Ep (AuNP_KG-37,-50 -90_ pretreated, 20 mg/L) cells, indicating neutralization of ethanol-induced oxidative stress by AuNP_KG_. Other AuNPs were variably effective, providing <20% protection. Unlike lipid peroxidase activities, LDH activities increased gradually and peaked at day 4. Then, a gradual decrease occurred. LDH activities in An/Ep cells and ApEp (AuNP_KG_ pretreated) cells did not differ significantly at days 1, 8 or 16, but differed significantly at days 2 and 4. Other AuNPs were variably effective, showing 5% to 30% increase.

#### 3.4.3. Enumeration of Apoptotic and Necrotic Cells

**Figure 12:**
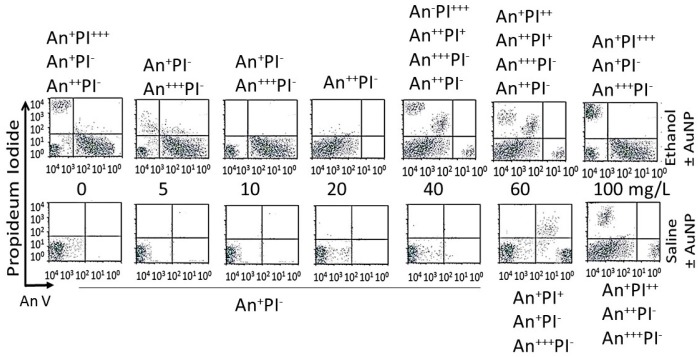
Effects of different doses of AuNPs on incorporation of annexin V and propidium iodide (PI) in An/En (bottom 0 mg/L), Ap/En (bottom 5 to 100 mg/L), Ap/Ep (top 5 to 100 mg/L AuNP) and An/Ep (top 0 mg/L) cells. Superscripts ^+^ to ^+++^ represent relative distribution of An and/or PI positive cells (^−^: An negative, ^+^: lowest distribution, ^++^: intermediate distribution and ^+++^: high distribution).

Dose–response (16-day data): Figure 12 shows distribution of different cell phenotypes in An/En, Ap/En (different AuNP dose) and Ap/Ep cells. An/En and Ap/En (5 to 40 mg/L AuNP dose) cells exhibited mostly An^−^PI^−^ phenotypes. Ap/En (60 mg/L AuNP dose) cells exhibited An^−^PI^−^ and An^+++^PI^−^ phenotypes, while Ap/En (100 mg/L AuNP dose) exhibited An^+^PI^−^, An^+++^PI^−^ and An^+^PI^++^ phenotypes. This suggests that 5 to 40 mg/L doses of AuNP_KG-50_ did not induced apoptosis or necrosis in cell cultures. The cell phenotypes in Ap/En cells exposed to other AuNPs are shown in Table 2.

An/Ep cells exhibited An^+^PI^−^, An^++^PI^−^ and An^+^PI^+++^ phenotypes. The Ap/Ep (AuNP_KG_ dose 5 and 10 mg/L) cells exhibited An^−^PI^−^ and An^++^PI^−^ phenotypes. Ap/Ep (AuNP_KG_ dose 20 mg/L) cells exhibited An^++^PI^−^ phenotypes, while Ap/Ep (AuNP_KG_ 60 and 100 mg/L) cells exhibited An^++^PI^−^, An^+++^PI^−^, An^++^PI^+^ and An^−^PI^+++^ phenotypes. The cell phenotypes in Ap/Ep cells exposed to other AuNPs are shown in Table 2.

**Figure 13:**
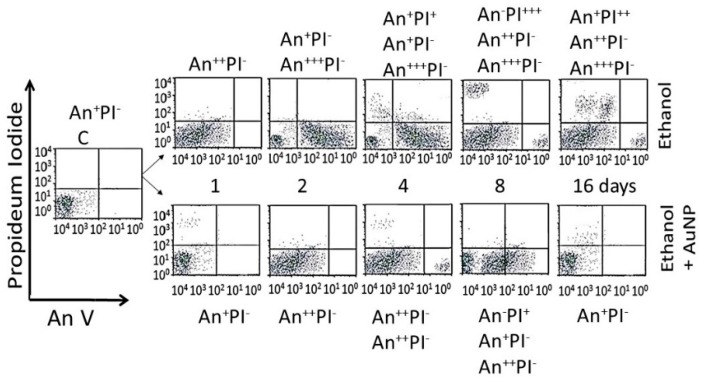
The time course of incorporation of annexin V and propidium iodide in An/En cells (C), Ap/En cells (bottom, 1 to 16 days, fixed AuNP dose of 20 mg/L), Ap/Ep cells (top, 1 to 16 days, fixed AuNP dose of 20 mg/L) and An/Ep cells (top day 1) cells. An: Annexin, PI: Propidium iodide, Superscripts: ^−^: within control range, ^+^: 10% to 15% increase, ^++^: >15% to 30% increase and ^+++^: >30% increase.

Time course (20 mg/L data): distribution of cell phenotypes at different time intervals after ethanol exposure in An/Ep and Ap/Ep (AuNP_KG_ dose 20 mg/L) cells are shown in Figure 13. At day 16, An/Ep cells exhibited An^+++^PI^−^, An^++^PI^−^, An^+^PI^++^ and An^−^PI^+++^ phenotypes, while Ap/Ep (AuNP_KG_ dose 20 mg/L) cells exhibited An^+/−^PI^−^ phenotypes, suggesting that a 20 mg/L dose of AuNP_KG-50_ protected against ethanol toxicity. The data for other NPs are not shown since they provided poor protection against ethanol toxicity. 

#### 3.4.4. NFκB Activation

**Figure 14:**
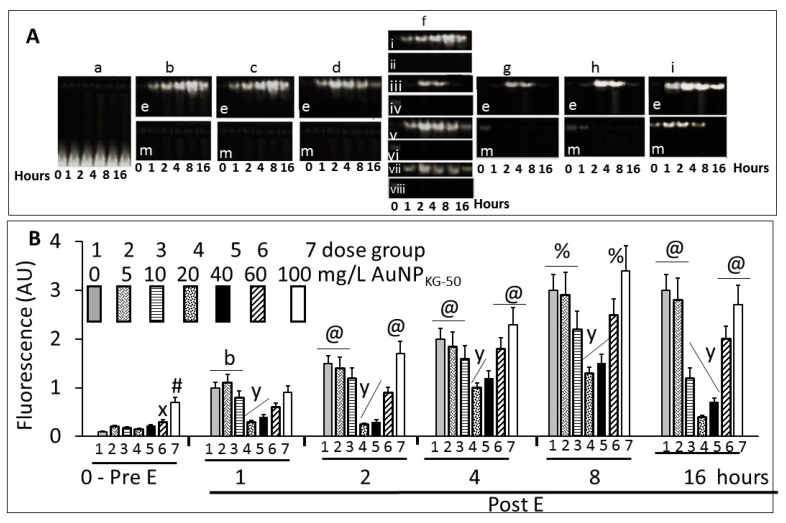
(**A**) Typical electrophoretic separation of NFκB dimer, p65–p50, in An/En (matrix), An/Ep (E+matrix), Ap/Ep (E+ 5 to 100 mg/L, data for AuNP_KG-50_ is shown), and Ap/En (saline + 5 to 100 mg/L, data for AuNP_KG-50_ is shown) cells. [a] matrix exposed control, An/En cells, [be] AuNP negative but ethanol positive An/Ep cells, [bm] AuNP and ethanol negative An/En cells, [ce] Ap/Ep cells receiving 5 mg/L AuNP_KG-50_, [cm] Ap/En cells receiving 5 mg/L AuNP_KG-50_, [de] Ap/Ep cells receiving 10 mg/L AuNP_KG-50_, [dm] Ap/En cells receiving 10 mg/L AuNP_KG-50_, [f] Ap/Ep cells receiving 20 mg/L AuNP_PEG+K+G_ (fi), AuNP_KG-50_, (fiii), AuNP_K-50_ (fv) and AuNP_G-50_ (fvii), and [ge, he, ie] Ap/Ep cells receiving 40, 60, and 100 mg/L, respectively, and *[gm, hm, im]* Ap/En cells receiving 40, 60, and 100 mg/L, respectively; (**B**) Scan of the p65–p50 fluorescence—dose–response and time course studies. (0 –Pre E) Ap/En cells were exposed to different doses of AuNP_KG-50_. (1 to 16 days post ethanol exposure): Ap/Ep cells were exposed to different doses of AuNP_KG-50_ for 4 days then ethanol for 24 h. Cells were analyzed at day 1, day 2, day 4, day 8 and day 16 post ethanol. Values are mean ± SD. x: *p* < 0.05 when values for cells exposed to 60 mg/L were compared with cells exposed to lower AuNP doses. #: *p* < 0.05 when values for cells exposed to 100 mg/L were compared with cells exposed to lower AuNP doses. b: *p* < 0.05 when values for cells exposed to 0, 5, and 10 mg/L were significantly higher than the values for cells exposed to higher doses. y: *p* < 0.05 when values for cells exposed to 20 and 40 mg/L were compared with other values. @: *p* < 0.05 when values for same AuNP dose collected at different time intervals were compared. Values are mean ± SD, *n* = 5.

Figure 14A shows typical gel images of NFκB’s (p65–p50)–DNA complex by the fluorescent EMSA. The specificity of the DNA-binding protein was confirmed by competition experiments in which the formation of protein–DNA complex was specifically in competition with a non-labeled specific probe, and by different amounts of nuclear proteins. Figure 14B shows fluorescence intensity of the NFκB–DNA band. In the absence of ethanol, a 5, 10, 20, or 40 mg/L dose of AuNP_KG-50_ did not modulate, while 60 mg/L and 100 mg/L dose significantly activated p65–p50. Ethanol exposure, in the absence of AuNPs, gradually increased NFκB activation that peaked at day 8. At day 16, the values either did not change, or decreased slightly. The 5 and 10 mg/L dose of AuNP_KG-50_ did not, while the 20 mg/L and 40 mg/L doses significantly attenuated NFκB activation by ethanol, thus protecting the cells against the ethanol’s adverse effects. Higher AuNP doses augmented NFκB activation, ensuing toxicity by ethanol.

#### 3.4.5. Formation of Intracellular Protein–Corona

**Figure 15:**
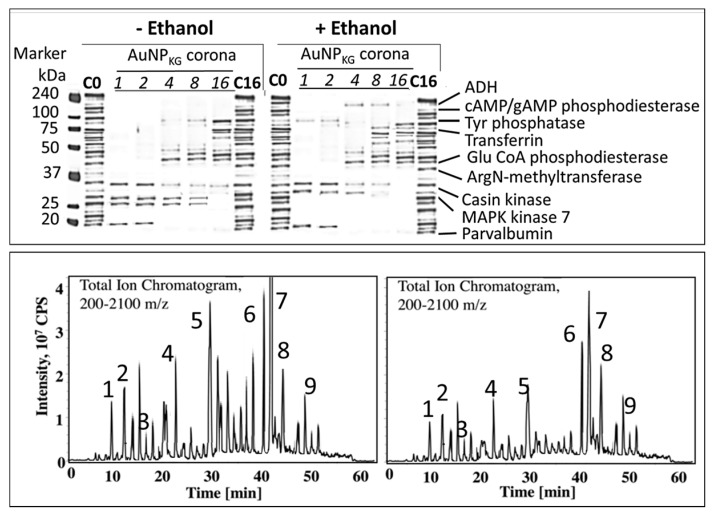
Characterization of protein corona on AuNP surface. (**Top**) Effects of ethanol exposure on composition of corona proteins. (**Bottom left)** Chromatographic separation of peptides extracted from ethanol-negative cells. **(Bottom right)** Chromatographic separation of peptides extracted from ethanol-positive cells. Protein identification: 1: alcohol dehydrogenase, 2: cAMP/cGMP phosphodiesterase, 3: Tyr phosphatase, 4: transferrin, 5: glutamate CoA phosphodiesterase, 6: arginine *N*-methyltransferase, 7: casein kinase, 8: MAPK kinase-9, and 10: parvalbumin. C_0_: control cells, C16: control cells exposed for 16 days; ADH: alcohol dehydrogenase; cAMP: cyclic adenosine dinucleotide mono phosphate; cGMP: cyclic guanine dinucleotide mono phosphate; MAPK: mitogen activated protein kinase; CPS: cAMP/gAMP phosphodiesterase.

**Figure 16:**
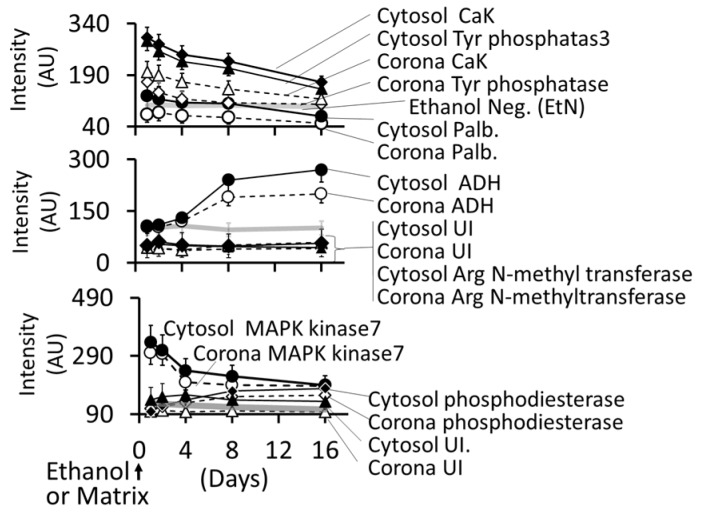
Time course of change in individual protein concentrations in cytosol and desorbed from AuNP_KG-50_. Abbreviation: CaK: casein kinase, Tyr: tyrosine, Palb: parvalbumin, UI: unidentified.

Figure 15 shows electrophoretic separation of cytosolic and AuNP–corona proteins from cells exposed to different AuNP preparations. The cytosol proteins ranged from 240 kDa to <10 kDa, while the AuNP corona proteins ranged from 100 kDa to <10 kDa in both control and ethanol-exposed cells. The protein composition exhibited the following differences (Figure 15 and Figure 16):

i Out of 35 proteins detected in control (An/En) cells, 9 cytosolic proteins were confirmed. Tyr phosphorylase, Arg N-methyl transferase, and MAPK kinase-7 yielded stronger bands (scan density 2.2 to 2.6) than parvalbumin, ADH, Glu-CoA dehydrogenase, cAMP/gAMP phosphodiesterase, transferrin, and casein kinase 1γ2 (scan density 0.3 to 1.4). Day 1 to day 16 values did not differ significantly.
ii The AuNP corona protein/cytosol protein ratio for Tyr phosphorylase, Arg *N*-methyl transferase, and MAPK kinase-7 increased from <10 at day 1 to >20 at day 16. The ratio for other proteins either did not change or increased slightly.
iii Ethanol exposure did not significantly alter the protein scan values, but significantly suppressed the AuNP corona protein/cytosol protein ratio. Ethanol specifically altered binding of MAPK kinase-7, casein kinase, Tyr phosphorylase and cAMP/gAMP phosphodiesterase to AuNPs, possibly by altering their concentrations in the cytosol. This suggests that ethanol exposure may have directly affected the AuNP–protein interaction.
iv The composition of protein corona changed temporally. AuNPs extracted from day 1 and day 2 An/En or An/Ep cells interacted with smaller (<37 kDa) proteins, while AuNPs extracted from day 4 to day 16 cells interacted with proteins ranging from <37 kDa to 150 kDa. The band intensity increases with increasing incubation time.

## 4. Discussion

The present study showed that aqueous extracts of kudzu root and edible gum provided a simple one-step synthesis of AuNPs that had many advantages over the traditional synthesis method. The AuNPs were well dispersed and retained the advantages, while minimizing the disadvantages of the medicinal plant extract. The following sections describe the characteristics and medicinal properties of AuNPs synthesized by kudzu root and gum extracts. 

### 4.1. Synthesis and Characterization of AuNPs

#### 4.1.1. AuNP Size, UV Absorption and FTIR Spectroscopy

The UV spectra of AuNPs indicated a sharp absorption maxima at around 526 to 586 nm, a characteristic absorbance for AuNPs, arising possibly due to an interaction of electromagnetic radiations with surface plasmon of AuNPs. Like the observations of Amendola and Meneghetti [41], the present study also showed that AuNPs synthesized in this study exhibited considerable differences around 200–586 nm, but no differences after the surface plasmon resonance (SPR) region around 500 to 600 nm [42]. In general, absorption maxima around 526 to 586 nm is determined by the AuNP size and incubation temperature. AuNPs of 22, 48, and 99 nm diameters, respectively exhibited absorption maxima at 540, 533, and 575 nm. Temperature affected UV absorption of AuNPs in a size dependent manner: smaller nanoparticles (<20 nm diameter) were relatively resistant to temperature changes, while an increase in size is associated with an increase in temperature sensitivity [43]. The present study indicated that AuNPs synthesized at different temperatures exhibited differences in the absorption maxima. More research is needed to explain this divergence.

Figure 3 show the FTIR spectra for gum extract, kudzu extract, and AuNP_KG-50_. Gum extract exhibits the absorption bands at (i) 3246 cm^−1^, possibly due to the O–H stretching band, (ii) 2901 cm^−1^ due to aliphatic C–H stretching, (iii) 1442, 1374, and 1339 cm^−1^ due to C–H bending vibrations, (iv) 1220 to 998 cm^−1^, due to C–H and O–H deformation, and (v) 1145 to 554 cm^−1^, due to the C–O and C–C groups’ vibration modes [44]. These bands characterize the carbohydrates present in gum solution. Kudzu extract exhibited a broad band between 3600 and 3000 cm^−1^ that is assigned to O–H stretching and to H-bonding involving the –OH groups. The band at 2920 cm^−1^ is assigned to CH_2_ symmetrical stretching vibrations. The band at 1639 cm^−1^ is attributed to the scissoring of two O–H bonds, while the bands at 750 cm^−1^ are due to skeletal stretching vibrations. The FTIR spectra for AuNP_KG-50_ (synthesized by kudzu–gum mixture at 50 °C) exhibited bands at 3306 cm^−1^ assigned to O–H stretching vibration of alcohols and phenols, 1630 cm^−1^ assigned to C=O stretching vibration of tertiary amides, 1206 cm^−1^ assigned to C–C–O stretching of epoxy rings, and 1044 cm^−1^ assigned to C=O stretching of primary alcohol [44]. From these observations, it is clear the polyphenols present in the extract are responsible for the reduction and stabilization (Figure 3). The observation that FTIR spectra for AuNP_KG-50_ contains bands that are also present in kudzu and gum extracts suggests that the many of the kudzu and gum ingredients are adsorbed onto the AuNP surface (Table 3).

#### 4.1.2. Characterization of Surface Ligands

An important observation of this study was that the two MS methods, LDI-TOF-MS and low matrix MALDI-TOF-MS, detected different sets of surfaces and ligands: low-matrix MALDI detected the AuNPs’ surface charge, while LDI-TOF-MS detected the desorbed organic ligands, including an internal standard added to the plant extract. The identities of the ligands were confirmed by analyzing a mixture of authentic standards by the same method. As shown in Figure 4, AuNP_KD-50_ contained the components of kudzu extract (daidzein, puerarin, and kakkasaponin III), gum (arabinose, galactose, and fructose) and the internal standard (d4-daidzein). The intensity of natural and d4 daidzein did not significantly change during 16 days of cell maintenance. The internal standard allowed semi-quantitative determination of ligand concentrations onto the AuNP surface. This allowed determination of ligand dose, as well as the extent of ligand depletion. Earlier studies have shown that puerarin, a naturally occurring isoflavone C-glycosides found in the roots of *Pueraria lobata* and *P. thomsonni*, are widely used in China and India for the treatment of alcoholism, cerebrovascular, and cardiovascular diseases [45]. Dietary gum exhibits anti-inflammatory and anti-oxidative properties [46]. Thus, the AuNP’s surface ligands may be responsible for beneficial effects reported for the phytochemical-synthesized AuNPs.

### 4.2. Cellular Uptake and Extrusion of AuNPs

**Figure 17:**
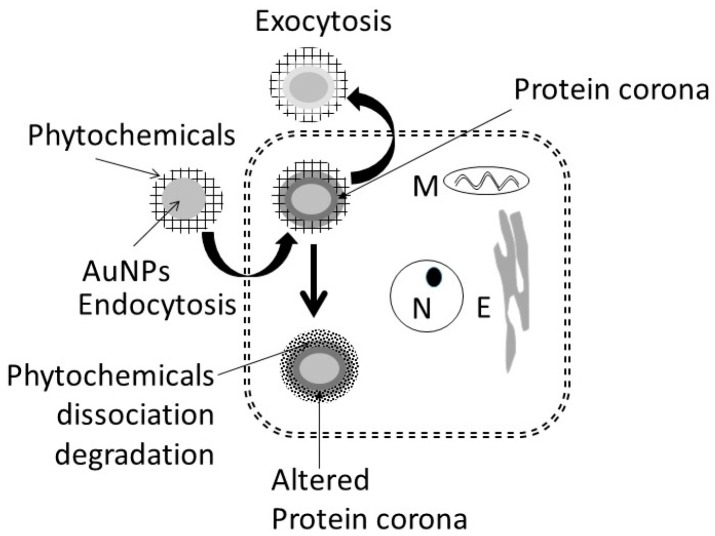
Possible mechanism proposed for uptake and accumulation of AuNPs in cells. N: nucleus, M: mitochondria, E: endoplasmic reticulum.

Figure 17 shows uptake, fate, and extrusion of AuNP in cells. NPs, upon encountering cell membranes, interact with (i) caveolin receptors targeted by small AuNPs (5 nm > 20 nm >> 50 nm); (ii) clathrin receptors for non-phagocytic uptake targeted by relatively larger AuNPs (50 nm) in the absence of serum; and (iii) scavenger receptor that mediates phagocytic uptake targeted by relatively larger AuNPs (50 nm) in the absence of serum [46,47,48,49,50]. 

Once internalized, NPs either enter the cytosol directly and interact with cytosol proteins [49] or are trapped in endosome–lysosomes complex for processing (NP defunctionalization, protein hydrolysis, and surface modification) before being transported to the cytosol and the cell’s periphery for extrusion via exocytosis [47,51,52,53]. In general, the endosomal NPs are extruded within 30 to 60 min [54], NPs accumulated in multi-vascular bodies exit cells within 6 days [55], and microtubule accumulated NPs are extruded constitutively via Golgi apparatus [56]. Internalization of nanoparticles can be modified by surface modification [57,58]. PEG coupling of nanoparticle surface significantly reduced the cytotoxic effects of nanoparticles in vitro and in vivo [59,60]. 

The present study showed that AuNP synthesized using different plant extracts and incubation temperatures exhibited differences in size (AuNP_KG_ > AuNP_K_ > AuNP_G_ or AuNP_PEG_) and composition of surface ligand. A time-dependent decrease in density of certain ligands were observed (Figure 7). In ethanol-negative cells, AuNP intake maxed within 3 days, and the intracellular NP concentrations exhibited the following patterns: AuNP_KG-90 and -50_ > AuNP_KG-37_, > AuNP_K-90,050,-37_ > AuNP_G_ or AuNP_PEG_ + K + G. After discontinuation of NPs, the iAuC decreased gradually and returned to basal levels within 16 days. Earlier studies have shown that AuNPs and other NPs are internalized by cells in a size- and surface-dependent manner through (i) caveolin-mediated uptake targeted by small AuNPs (5 nm > 20 nm >> 50 nm); (ii) clathrin-mediated non-phagocytic uptake targeted by relatively larger AuNPs (50 nm) in the absence of serum; and (iii) scavenger-receptor mediated phagocytic uptake targeted by relatively larger AuNPs (50 nm) in the absence of serum [46,47,48,49,50]. Since SH-SY5Y are non-phagocytic, they may be internalized via caveolin-mediated and clathrin-mediated endocytosis. This suggests that, unlike the AuNPs’ uptake that varied according to the synthesis procedure, their excretion from the cells did not differ significantly.

In ethanol-positive cells, harvested at 1, 2, 4, and 8 days after ethanol discontinuation, iAuCs were either significantly lower than (as for AuNP_KG_ and AuNP_PEG_ pretreated cells) or comparable to (as for AuNP_K_ or AuNP_G_ pretreated cells) the corresponding values in ethanol-negative cells. At day 16, the two cell groups exhibited comparable values. This suggests that ethanol may differentially affect internalization of AuNPs that may be associated with unique properties of ethanol. Earlier studies have shown that ethanol, due to its polar and nonpolar characteristics, can (i) leak through the cell membrane and form hydrogen bonds with phospholipid head groups that disrupts the hydrophobic chain orientation, and (ii) increases the membrane fluidity by denaturing proteins and affecting the lipids, resulting in formation of small holes in the membrane [50,61,62]. Ethanol, by forming H-bonds, may orient the phospholipids outwards, thus increasing their movement and the membrane fluidity increases and affecting the integrity of the membrane receptors [63]. Therefore, AuNP_KG_, being relatively smaller nanoparticles, may escape the cells via exiting through the ethanol-induced membrane holes. 

### 4.3. Toxicity of Plant-Extract Synthesized AuNPs

Although surface functionalized AuNPs, because of their unique properties, have emerged as promising candidates for biomedical applications, such as sensing, imaging, and drug delivery [64], their application in clinical setting has not materialized, due to uncertainties in their adverse effects. Since the NP toxicity is impacted by their size, shape, charge, and surface functionalization, toxicity of each individual NP preparation must be evaluated before testing the beneficial effects. The present study showed that a 5 to 20 mg/L dose of AuNP preparations did not significantly affect (i) viable cell enumeration; (ii) LDH and lipid peroxidase activities; (iii) NFκB activation, and (iv) apoptotic/necrotic cell enumerations. However, 40 to 100 mg/L dose caused a dose-dependent increase in these indices. This suggests that relatively higher doses (>60 mg/L) of AuNPs may cause different degrees of cytotoxicity in SH-SY5Y cells. These results are consistent with previous investigations performed with MCF-7 (human breast adenocarcinoma cell line 7), PC-3 (prostate cancer cell line 3), and dermal fibroblasts [65,66] which demonstrated that the AuNPs, at higher doses (>100 mg/L), impaired the proliferation of dermal fibroblasts, and induced an abnormal formation of actin filaments, causing, therefore, a reduced cellular motility and influencing the cell morphology. Vijayakumar and Ganesan [67] reported that citrated and biotinylated 18 nm gold nanoparticles did not induce toxicity, while smaller particles were much more toxic in leukemic K562 cell lines. Similar results were reported by Cho et al. [68] in adeno-carcinomic human alveolar basal epithelial cell line, A549. These observations suggest that a 5 to 20 mg/L dose of AuNPs can be used safely in this experimental setup. However, toxicity must be reevaluated when the NPs physicochemical properties or the experimental conditions change.

### 4.4. Therapeutic Potency of Plant-Extract Synthesized AuNPs against Ethanol Toxicity 

#### 4.4.1. Ethanol Cytotoxicity

**Figure 18:**
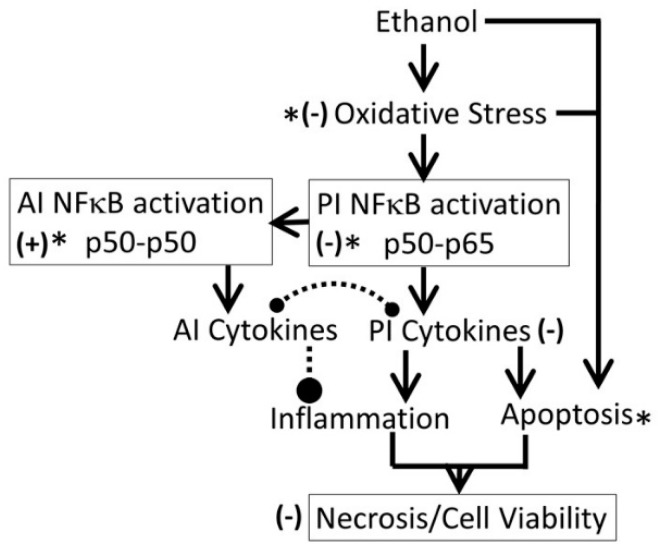
Proposed mechanism for ethanol-induced cytotoxicity. *: proposed target sites for AuNP’s protective effects. Solid lines: activation, broken lines: inhibition, (−): inhibitory effects of AuNPs and (+): activation by AuNPs. AI: anti-inflammatory, PI: pro-inflammatory.

It is well established that acute or chronic ethanol exposure (1) upregulated oxidative stress, and dysregulated inflammation and cell signaling [20]; (2) upregulated expression of cytokines, increased amounts of TNF-α, and depleted both cytosolic and mitochondrial GSH that may contribute to an increase in oxidative stress [69]; (3) significantly inhibited activities of p450 enzymes CYP1A1, CYP2B6, CYP2C19, and CYP2D6, that may alter xenobiotic metabolism and drug pharmacokinetics [70]; and (4) significantly reduced total number of embryonic stem cells, inducing the formation of cardiomyocytes [62]. The present study demonstrated that 54 mM of ethanol exposure exerted immediate cytotoxicity in SH-SY5Y cells, possibly via the mechanisms listed above. The cytotoxic effects of ethanol persisted for up to 16 days after the cells were treated with ethanol, suggesting that 24 h ethanol exposure may have long-term cytotoxicity, possibly dysregulating oxidative stress and inflammation (Figure 18). 

#### 4.4.2. Protective Effects of AuNPs against Ethanol Toxicity

This study showed that AuNPs synthesized using kudzu and gum extracts protected SH-SY5Y cells against ethanol toxicity, although to different degrees (AuNP_KG-90,-50_: 60 to 80% protection, AuNP_KG-37_, AuNP_K-90,-50,-37_: 30 to 50% protection, AuNP_PEG_ + K + G (20 to 30% protection), and AuNP_PEG_ and AuNP_G-50,-90_ (<20% protection). The present observations are supported by earlier experimental studies showing that many medicinal plants possessed preventive and therapeutic properties against alcoholism, alcohol withdrawal symptoms and alcohol toxicity [22,25,28,29,71,72,73,74,75,76,77,78,79,80]. Earlier studies have shown that high isoflavone contents in kudzu root, *Solanum torvum* fruit, *Cassia tora* leaves, *Atrocarpus altilis* leaves, and *Angelicae pubescens* extracts may be attributed to their protective effects against ethanol toxicity in vitro [81,82,83,84,85]. Despite strong evidence, clinical applications of phytochemicals are hindered by the following major hurdles: (i) poor solubility and bioavailability; (ii) inability to cross the blood–brain barrier; and (iii) lack of plant extract standardization that prohibits accurate determination of dose–response relationships, yielding high variability in results [86]. One of the key problems associated with earlier studies showing protective effects of plant-extract synthesized NPs is that they did not identify the NPs’ surface ligands, and thus, could not conduct dose–response studies. In the present study, LDI (no matrix) or low-matrix MALDI identified the ligands adsorbed onto the NPs surface. Inclusion of an internal standard allowed standardization of the NPs, and provides the possibility of conducting dose–response studies for surface ligands. 

#### 4.4.3. Possible Mechanisms for Protection against Ethanol

The protective effects of plant-extract synthesized AuNPs may be attributed to the adsorption of bioactive compounds onto the AuNPs surface. In the present study, the following ligands have been identified from the AuNPs surface (Figure 4):

(i) Gum ligands, such as arabinose, galactose, and fructose, that are part of a highly branched polysaccharide (MW 3 × 10^5^) consisting of b-(1-3) galactose backbone with linked branches of arabinose and rhamnose [87]. Ali et al. [35] have provided direct evidence of anti-inflammatory and anti-oxidative capacities of edible gum Arabic (GA) that ameliorates superoxide production and DNA double strand breakage. Cuesta et al. [88] and Faggio et al. [89] have reported high immune-stimulatory, anti-inflammatory and anti-oxidation properties of GA in mammals and aquatic animals, respectively. Studies [90,91,92,93] have also shown that edible gum, in addition to being anti-oxidative, enhance the biocompatibility and bioavailability of AuNPs and other NPs, such as AgNPs and magnetic iron oxide nanoparticles (MNP). The GA coating offers two major benefits: it enhances colloidal stability and provides reactive functional groups suitable for coupling of bioactive compounds.
(ii) Kudzu ligands, such as daidzein and puerarin possess potent anti-inflammatory and anti-oxidation properties, thus protecting against alcohol-mediated disorders [28,29,94,95,96,97]. As shown in Figure 10, ethanol induces oxidative stress and ensuing downstream activation of p50–p65 (a pro-inflammatory dimer of NFκB) and suppression of p50–p50 (an anti-inflammatory dimer of NFκB), resulting in dysregulation of inflammation and activation of pro-apoptosis signaling [32]. The addictive effects of ethanol are mediated by the brain addiction pathways including dopamine, GABA, NMDA, and serotonin neurons [5,98,99]. Earlier studies have shown that kudzu root extracts modulate the brain addiction circuits, especially the dopamine, GABA, NMDA, and Glu neurons/receptors in protecting the brain against the adverse effects of ethanol and suppressing the severity of the withdrawal symptoms [100,101,102].

Taken together, these observations suggest that pretreatment of cells with the AuNP-adsorbed plant components are more potent in protecting the cells against ethanol toxicity than pretreatment of cells with crude plant extracts their purified components. 

#### 4.4.4. AuNP–Protein Interaction

It is well established that NPs in biological and environmental media (blood, intracellular fluid, plant extracts, or environmental samples) interact with the media components such as soluble proteins, lipids and other ingredients, and form a surface corona that may modulate the NPs’ physicochemical and biological (cellular/tissue responses such as cellular uptake, kinetics, signaling, accumulation, transport, and toxicity) properties [103,104,105,106,107,108]. Formation of protein corona in blood or intracellular fluids may depend upon the composition and organization of surface proteins [109]. In general, the corona may be divided into two components: the “hard” corona with long exchange time, and “soft” coronas with short exchange times [109]. In a biological fluid, the surface corona’s protein composition, amount, and presentation on the surface, modifies their beneficial, therapeutic or adverse effects [110,111,112,113,114,115]. In the present study, nine proteins, ranging from <10 to 200 kDa, have been identified in cytosol and hard corona of AuNPs synthesized using different plant extracts and incubation temperature (Figure 14A). AuNP_KG-50_ (average diameter ~8 nm), AuNP_K-50_ (average diameter ~39 nm), and AuNP_G-50_ (average diameter ~41) interacted with protein <37 kDa, 50 to 37 kDa, and >75 kDa, respectively. This suggests that the size and surface ligand composition may determine the composition of proteins’ corona. As shown in Figure 14B, the time course of change in individual corona proteins paralleled the change in corresponding cytosol proteins, suggesting the possibility of rapid exchange between corona and cytosol. It is hypothesized that poor protein-binding capacity of AuNP_KG-50_ may contribute to its high therapeutic activity and relatively lower toxicity.

## 5. Conclusions

Although kudzu extract, edible gum extract and a combination of both (AuNP_KG-37,-50,-90_) incubated at 37 °C, 50 °C, and 75 °C, reduced Au^3+^ into colloidal gold nanoparticles (kudzu synthesized: AuNP_K-50,-90,-37_, gum synthesized: AuNP_G-50,G-90_, and kudzu+gum synthesized: AuNP_KG-37,-50,-90_ ), they differed in size (AuNP_KG_ << AuNP_K_ < AuNP_G_) and physicochemical properties, including their interaction with cytosol proteins (AuNP_KG_ << AuNP_K_ < AuNP_G_). The surface of each AuNP contained the extract’s active ingredients that were analyzed and confirmed using LDI and low-matrix MALDI. AuNP_KG-50_ was least toxic to SH-SY5Y cells and most effective in suppressing the adverse effects of ethanol on SH-SY5Y cells. The beneficial and adverse effects of AuNPs may have been modified by the formation of proteins corona.

## Figures and Tables

**Table 1:** List of fragment ions monitored for protein identification.

Protein	Test: IS or MS: Sequence	*m*/*z* (+1)	*m*/*z* (+2)
Bin1 protein	MS: AEEELIK	832.2	418.1
Casein Kinase 1 γ2	MS: MEYVHTK	908.0	456.3
cAMP/cGMP phosphodiesterase	MS: VOYHNWK	944.4	-
Glutaryl-Co-A dehydrogenase	MS: CEDNCIR	853.3	439.1
Arginine *N*-methyltransferase	MS: QYKDYK	845.5	424.3
Lactate dehydrogenase	MS: MVSGESR	766.52	384.3
MAPK kinase 7	MS: LCDFGISGR	968.7	-
Parvalbumin	MS: FFQMVGLK	970.5	486.4
Protein Tyr Phosphatase	MS: MPVIVSR	803.1	-
Transferrin	MS: EDPQTFYY	815.5	209.3
Triose phosphate isomerase	MS: AISDNVK	731.4	-

IS: internal standard; MS: mass spectrum; cAMP: cyclic adenosine dinucleotide mono phosphate; cGMP: cyclic guanine dinucleotide mono phosphate; MAPK: mitogen activated protein kinase.

**Table 2:** Effects of AuNP dose on SH-SY5Y cell phenotype.

NP	0	5	10	20	40	60	100
An/En cells (no AuNPs or ethanol)							
-	An^−^PI^−^	-	-	-	-	-	-
Ap/En cells (AuNPs positive but ethanol negative)							
AuNP_KG-90,50,37_ AuNP_K-90,50,37_	-	An^−^PI^−^	An^−^PI^−^	An^−^PI^−^	An^−^PI^−^	An^++^PI^−^	An^−^PI^−^ An^++^PI^+^ An^+++^PI^+^
AuNP_G-50,90_	-	An^+^PI^−^	An^+^PI^−^	An^+^PI^−^	An^+^PI^−^	An^+^PI^−^	An^−^PI^−^ An^+++^PI^−^ An^+^PI^++^
AuNP_PEG, PEG+K+G_	-	An^+^PI^−^	An^+^PI^−^	An^l+^PI^−^	An^++^PI^−^	An^+++^PI^−^ An^++^PI^−^ An^+^PI^+^	An^+^PI^−^ An^+++^PI^+^ An^−^PI^++^
An/Ep cells (AuNPs negative, but ethanol positive)							
	An^−^PI^+++^ An^++^PI^−^ An^+^PI^++^ An^+++^PI^−^	^−^	-	-	-	-	-
Ap/Ep cells (AuNPs and ethanol positive)							
AuNP_KG-90,-50,-37_	-	An^−^PI^−^ An^++^PI^−^ An^++^PI^+^	An^++^PI^−^	An^++^PI^−^	An^++^PI^−^	An^+^PI^−^ An^+^PI^+^ An^++^PI^+^	An^−^PI^−^ An^++^PI^+^ An^−^PI^++^
AuNP_K-90,-50,-37_	-	An^−^PI^−^ An^++^PI^−^ An^++^PI^+^	An^−^PI^−^ An^+^PI^+^	An^+^PI^−^ An^+^PI^++^	An^+^PI^−^ An^+^PI^++^	An^−^PI^−^ An^++^PI^+^	An^−^PI^+^ An^++^PI^−^ An^++^PI^++^
AuNP_G-50,-90_	-	An^−^PI^−^ An^+^PI^++^	An^+^PI^−^ An^+^PI^++^	An^+^PI^−^ An^+^PI^+^	An^+^PI^−^ An^++^PI^+^ An^+^PI^+^	An^+^PI^−^ An^++^PI^+^ An^−^PI^+^	An^−^PI^+^ An^++^PI^−^ An^++^PI^+^
AuNP_PEG,PEG+K+G_	-	An^++^PI^−^	An^++^PI^−^ An^++^PI^+^	An^++^PI^−^ An^+^PI^++^	An^++^PI^−^ An^++^PI^+^ An^−^PI^++^	An^++^PI^−^ An^++^PI^+^ An^−^PI^++^	An^−^PI^−^ An^++^PI^−^ An^++^PI^+^ An^−^PI^+++^

Superscripts ^–, +, ++, +++^ show relative distribution of An and/or PI positive cells as described in Figure 12.

**Table 3:** Description of FTIR peaks.

Peak cm^−1^	Functional Group/Vibration Mode
750	Aromatic protons
927	C–O–C vibration modes of α-1,4 glycosidic linkage
1040/1162	Carbonyl (C=O) group, C–O stretching alcohols (primary, secondary and tertiary), carboxylic acids, esters, and ethers
1020/1079 1116/1156	C–O/C stretching in exo/endo-cyclic bonds and C–O–H/C deformation modes of oligo/polysaccharides and absorption bands for carbohydrates
1383/1456	The spectral bands for C–O–H stretching in pyranose and C–H deformation in esters
1621/1637	Aromatic C=C/C–OH stretching in exocyclic bond
2923	Glucoside ring due to the stretching vibration of O–H [44]
3420	O–H, as also the H-bonded alcohols and phenols, carboxylic acid
